# Noxious and Fatal Effects of Septic Gas on a Family Living near the Place of Its Evolution

**Published:** 1807-03

**Authors:** John Henderson

**Affiliations:** Huntingdon, America


### *Noxious and fatal* EFFECTS *of* SEPTIC GAS *on a Family living near the Place of its Evolution: By Dr.* JOHN HENDERSON, *of Huntingdon, America.*

ON the 7th of September, 1805, I was called to visit a poor family of the name of Seyer, situate about twelve miles from this place, on Mill Creek, a small stream that empties itself into the Juniata river, and which, at that time, was very low, and emitted effluvia so disagreeable in the neighbourhood of the dwelling-house, as to produce nausea and disgust in all those who were exposed to it.

Upon inquiry I found that the eldest of the children had sunk under its baneful effects early in the season; and the next eldest, a young man, eighteen years of age, had been attacked with the common autumnal fever about six weeks previous to my visit, which had been suffered to take its course, uninterrupted by medicine, and that for two weeks past he had laboured under a severe diarrhœa, which had reduced him to the appearance of a skeleton; little or no attention had been paid to cleanliness of person or bedding, and, at this time, he might literally be said to be incrusted with his own excrements. About a week previous to this period, the three next eldest of the children had been attacked with fever of the typhus kind, attended with frequent chills for the first twenty-four hours, a weak quick pulse, great prostration of strength, a trifling cough, nausea, and inclination to vomit, fetid diarrhœa, frequent bleeding at the nose, and in one of them delirium from the first appearance of disease. In a few days subsequent to my first visit, every member of the family (Mr. Seyer excepted), amounting to twelve in number, was seized with this direful disease; the five eldest of the children soon sunk under its violence; the remainder, after lingering an indefinite length of time, recovered very slowly.

There

There were some phenomena in the progress of this disease so extraordinary, and out of the common course of nature, which I had never before met with in my practice, that they excited a great deal of speculation in those who were witnesses, and in others throughout this country, respecting the cause to which they should be attributed, and from their rare occurrence I think ought to be recorded. Owing to the extreme relaxation of the cutaneous vessels, and the great tenuity of the *blood*, in three of those to whom the disease proved fatal, for several days previous to their decease, this fluid oozed out from the extremities of the vessels through the cuticle, and stood in minute drops all over the face, chest, arms, &c. and when wiped off, no traces from whence it issued could be discovered by the closest inspection; but after death, a small livid spot appeared wherever there had been any exudation of the sanguineous fluid.

I felt no hesitation in ascribing the disease to the operation of *septic gas*, generated from the putrid animal excretions which surrounded and enveloped the eldest son, and which became so virulent in a short time after most of the family were confined, that it never failed, on my going into the house, to produce an increased flow of saliva, and excite a disagreeable taste, which I thought much resembled the impression made on the throat and fauces by tartar emetic.

In the treatment of this fever, I attempted to follow Dr. Mitchell's method of alkalizing, and diluting the venomous cause, by exhibiting carbonate of pot-ash, lime-washing the walls, even the floor of the house, ventilation, washing the bodies of the sick and their bedding with soap and water; but through the want of out-houses and nurses, and owing to the terror spread throughout the neighbourhood, it was impossible to have them separated, so that they were all obliged to lie in one common room. To some, in the commencement of the fever, where nausea and inclination to vomit attended, I gave emetics; to others, purgatives of jalap and calomel; and afterwards, in all, I endeavoured to charge the system with mercury as soon as possible, which the highly irritable state of the stomach and intestines rendered difficult; in the latter stages of the fever, I administered alkalies, combined with the cinchona, either in substance or decoction, as the stomach would bear it; gave wine freely, and made a liberal use of blisters and sinapsisms.

*April* 24, 1806.

